# Laser therapy treatment of phacomatosis pigmentovascularis type II: two case reports

**DOI:** 10.1186/1752-1947-7-55

**Published:** 2013-02-27

**Authors:** Koji Adachi, Shinji Togashi, Kaoru Sasaki, Mitsuru Sekido

**Affiliations:** 1 Department of Plastic and Reconstructive Surgery, Institute of Clinical Medicine, Graduate School of Comprehensive Human Science, University of Tsukuba, 1-1-1 Tennodai, Tsukuba, Ibaraki 305-8575, Japan

**Keywords:** Cutis marmorata telangiectatica congenita, Laser therapy, Phacomatosis pigmentovascularis

## Abstract

**Introduction:**

Phacomatosis pigmentovascularis is a rare congenital condition characterized by vascular malformation associated with extensive pigmented nevi. Even though it forms a large, prominent skin lesion, therapy for phacomatosis pigmentovascularis is rarely discussed. To the best of our knowledge, this is the first report of phacomatosis pigmentovascularis type II treated with combined laser therapy using Q-switched alexandrite and long-pulsed dye lasers.

**Case presentations:**

In the first of two cases reported here, a 2-week-old Japanese baby girl was given a diagnosis of phacomatosis pigmentovascularis type II and Klippel–Trénaunay syndrome because of port-wine stains, cutis marmorata telangiectatica congenita, and aberrant Mongolian spots over her trunk and limbs. After five laser therapy sessions under general anesthesia, her aberrant Mongolian spots and port-wine stains have improved. But interestingly, the cutis marmorata telangiectatica congenita on the patient's back has improved without laser therapy.

In the second case, a 4-month-old Japanese baby boy was referred to us because of port-wine stains, cutis marmorata telangiectatica congenita, and aberrant Mongolian spots over his face, trunk and limbs. Phacomatosis pigmentovascularis type II was diagnosed and laser therapy was started. After three laser therapy sessions under general anesthesia, the aberrant Mongolian spots and port-wine stains have improved. The cutis marmorata telangiectatica congenita on the baby's back, buttocks, and arms has faded somewhat without laser therapy.

**Conclusions:**

Combined laser therapy improved the phacomatosis pigmentovascularis skin lesions, but was not effective for the cutis marmorata telangiectatica congenita with hemiatrophy. Cutis marmorata telangiectatica congenita without atrophy can be expected to improve on its own. Our results will assist physicians considering how best to treat patients with phacomatosis pigmentovascularis.

## Introduction

Phacomatosis pigmentovascularis (PPV), a rare congenital condition, was first reported by Ota et al. in 1947 as a vascular malformation associated with extensive pigmented nevi [1]. PPV is classified into four types, each of which is further classified into two subtypes according to whether there is systemic involvement [2]. Although PPV skin lesions are quite large and can be emotionally distressing for patients and their families, therapy for PPV is rarely discussed. We here present two patients with PPV type II who were treated with combined Q-switched alexandrite laser (QAL) and long-pulsed dye laser (LPDL) therapy.

## Case presentations

### Case 1

A 2-week-old baby girl born to nonconsanguineous Japanese parents was referred to us because of skin lesions. The family history and personal history were unremarkable, and the pregnancy and labor had been uneventful. The baby's weight and height at birth were in the 50th percentile. On physical examination, we noted erythematous macules and plaques over her right chest, abdomen, and arm. We diagnosed these lesions as a port-wine stain (PWS). We also noted extensive areas of reddish-blue reticulated, marble-like lesions over her back and buttocks, consistent with a diagnosis of cutis marmorata telangiectatica congenita (CMTC). CMTC with hemihypertrophy was present on her lower right leg. Bilateral, demarcated greyish-blue hyperpigmentation on the buttocks and thighs, partly intermingled with the vascular lesions, was diagnosed as aberrant Mongolian spots. The rest of the physical examination was normal. Blood biochemistry, complete blood cell count, chest radiography, electrocardiography, and echocardiography were all within normal limits.

The physical findings indicated a diagnosis of PPV type IIb and Klippel–Trénaunay syndrome. Laser therapy was started when the patient was one-year-old. We used a QAL (ALEXLASER; Candela Corporation, Wayland, MA, USA) with an energy density of 6.0J/cm^2^ to treat the dermal melanosis of her buttocks and thighs. An LPDL (Vbeam®; Candela Corporation) with an energy density of 10J/cm^2^ and 1.5ms was used simultaneously to treat the PWS and CMTC lesions. Where the lesions were intermingled, we treated the dermal melanosis before the PWS and CMTC. The patient underwent five laser therapy sessions under general anesthesia. In each session, approximately 30% of the lesion area was treated; however, her back has yet to be treated.

**Figure 1:**
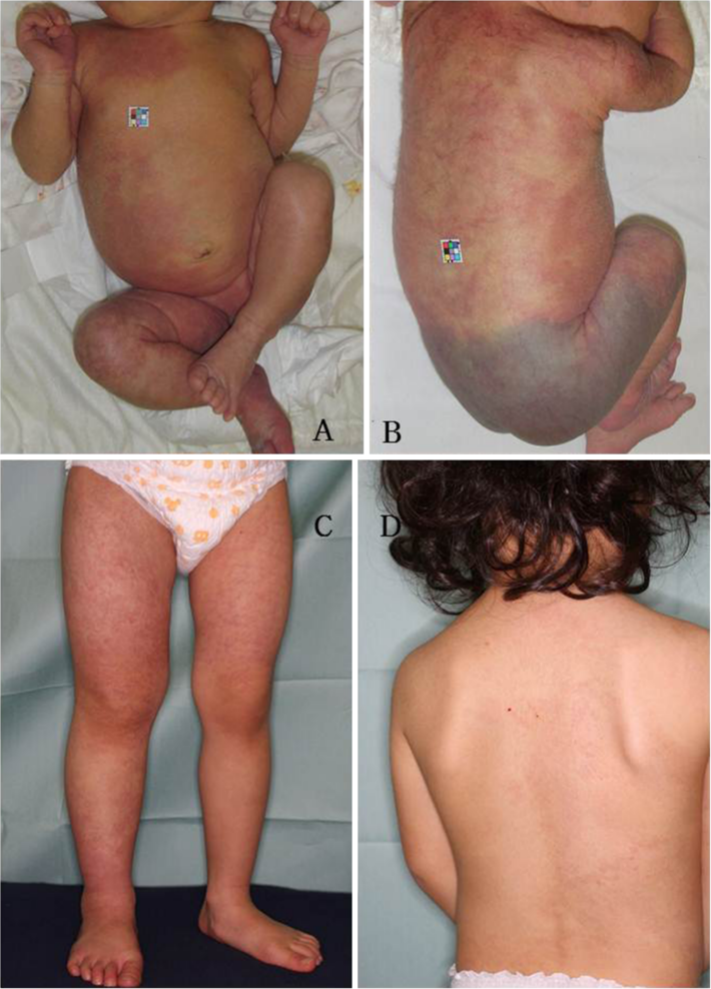
**Case 1: A baby with phacomatosis pigmentovascularis type IIb treated with combination laser therapy.** (**A**, **B**) The baby had a port-wine stain on her right side, hemihypertrophy of her legs, and cutis marmorata telangiectatica congenita (CMTC) on her back and legs. (**C**, **D**) In photographs taken after five sessions of laser therapy, the aberrant Mongolian spots showed improvement, but the CMTC remained mostly unchanged. The CMTC on the child's back showed improvement without laser therapy.

The patient is now 3-years-old and shows normal mental and physical development. Although the hemihypertrophy remains, there is no leg length discrepancy. The aberrant Mongolian spots have faded. The four authors of this paper evaluated the amount of fading and agreed that although the lesions are still visible their color has decreased by about 80%. The PWS on the patient's trunk and arm has also decreased by about 80%, but the CMTC on her leg is mostly unchanged. We plan to change the laser power and pulse width for this patient's future treatment. Of interest, the CMTC on the patient's back has faded by about 90%, without laser therapy (Figure 1).

### Case 2

A 4-month-old baby boy born to nonconsanguineous Japanese parents was referred to us because of skin lesions. The family history and personal history were unremarkable, and the pregnancy and labor had been uneventful. The boy's weight and height at birth were in the 50th percentile, and his postnatal development was normal. On physical examination, we noted a PWS on his right cheek and CMTC on his right side on his back, buttock, and arm. We found aberrant Mongolian spots, partly intermingled with the vascular lesions, on his chest and arms. We also noted ocular pigmentation of his right eye. Ophthalmologic examination revealed no other findings. No atrophy or hypertrophy of the soft tissues was observed. The rest of the physical examination was normal. Blood biochemistry, complete blood cell count, chest radiography, and electrocardiography were within normal limits.

PPV type IIa was diagnosed, and laser therapy began when the patient reached one year of age. We used a QAL with an energy density of 6.0J/cm^2^ to treat the dermal melanosis of his chest and upper limbs. We used an LPDL with an energy density of 10J/cm^2^ and 1.5ms to treat his PWS lesions simultaneously. Where the lesions were intermingled, we treated the dermal melanosis before the PWS. The patient underwent three laser therapy sessions under general anesthesia. We did not treat the CMTC in any of the sessions.

**Figure 2:**
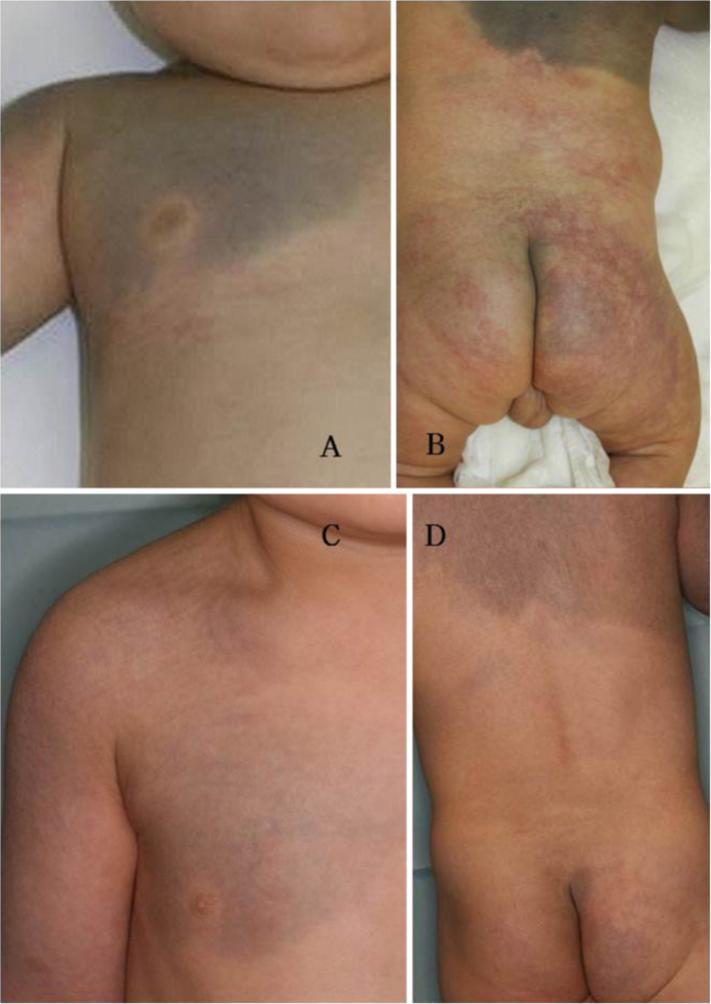
**Case 2: A baby with phacomatosis pigmentovascularis type IIa treated with combination laser therapy.** (**A**, **B**) The baby had aberrant Mongolian spots on his chest and arms, a port-wine stain (PWS) on his cheek, and cutis marmorata telangiectatica congenita (CMTC) on his lower back and buttocks. (**C**, **D**) Photographs taken after three sessions of laser therapy show improvement in the aberrant Mongolian spots and PWS. The CMTC improved without laser therapy.

The boy is now 2-years-old and shows normal mental and physical development. Both the aberrant Mongolian spots on his trunk and upper limb and the PWS on his cheek have decreased by about 60%, although they are still visible. We plan to treat this patient with another session of combined laser therapy. The CMTC on the child's back, buttocks, and arms has faded by about 90% without laser therapy (Figure 2).

## Discussion

PPV is classified into the following four types, according to the epidermal component accompanying the capillary malformation: type I, nevus flammeus (another term for PWS) with nevus pigmentosus et verrucosus; type II, nevus flammeus with aberrant Mongolian spots, with or without nevus anemicus; type III, nevus flammeus with nevus spilus or giant speckled lentiginous nevus, with or without nevus anemicus; and type IV, nevus flammeus with both aberrant Mongolian spots and speckled lentiginous nevus, with or without nevus anemicus [2]. Each type is subdivided as having (a) only oculocutaneous involvement or (b) extracutaneous features. In addition, there are reports of patients with CMTC and aberrant Mongolian spots, and the term PPV type V has been proposed for this condition [3,4].

In 2005, Happle proposed classifying PPV into three distinct categories [5], descriptively named phacomatosis cesioflammea, phacomatosis spilorosea, and phacomatosis cesiomarmorata. Our patients would be classified as having PPV IIa and IIb in the traditional classification system, and as having phacomatosis cesioflammea in the new classification system.

It has been proposed that PPV is the result of non-allelic twin spotting involving two different recessive mutations [6]. Postzygotic recombination in early embryogenesis produces two homozygous daughter cell lines that migrate to different parts of the body and form a mosaic pattern of capillary and melanocytic lesions.

PPV has a roughly 50% association rate with systemic disease in all categories [7]; Klippel–Trénaunay syndrome and Sturge–Weber syndrome are the most common identifiable disorders. Case 1 was classified as PPV IIb because of the presence of nevus flammeus, aberrant Mongolian spots, and Klippel–Trénaunay syndrome.

Although PPV skin lesions can be distressing for patients and their families, very few attempts have been made to treat PPV with laser therapy. Ono and Tateshita reported an improvement in one case of PPV treated with a Q-switched ruby laser and a dye laser [8]. Kono *et al*. suggested a combined laser approach (a Q-switched ruby laser, a QAL, and a flashlamp-pumped pulsed-dye laser) for PPV [9]. It has been reported that aberrant Mongolian spots can be effectively treated with Q-switched ruby and alexandrite lasers [10], and that PWS can be treated safely and effectively with LPDL [11]. Therefore, we used QAL and LPDL for PPV therapy.

Laser treatment of intermingled vascular and pigmented lesions can be difficult. Thus, we recommend beginning PPV therapy with the QAL, followed by the LPDL after the dermal melanin content has subsided to some extent [8].

Mazereeuw-Hautier *et al*. reported that frequency-doubled Nd:YAG (neodymium-doped yttrium aluminum garnet; Nd:Y3Al5O12) laser therapy failed to improve CMTC because of dilated veins and extensive large, deep capillaries [12]. For the same reason, LPDL therapy was not effective for our first patient's CMTC with hemiatrophy. The power and pulse width will be raised for this patient's next laser therapy session.

Because CMTC without atrophy can be expected to improve on its own, vascular lesions should be examined carefully to differentiate between PWS and CMTC and to ascertain whether atrophy is present.

## Conclusions

We have reported two cases of PPV type II treated with combined laser therapy. The combined laser therapy improved the PPV skin lesions, the PWS, and aberrant Mongolian spots, but was not effective for CMTC with hemiatrophy. However, CMTC without atrophy can be expected to improve on its own. To the best of our knowledge, this is the first report of PPV type II treated with combined QAL and LPDL laser therapy. Our results will be of some help to physicians in deciding how best to treat patients with PPV.

## Consent

Informed written consent was obtained from the parents of both patients to publish this case report and the accompanying images; copies of the written consents are available for review by the Editor-in-Chief of this journal.

## Competing interests

The authors declare that they have no competing interests.

## Authors’ contributions

KA performed the diagnosis and laser therapy, reviewed the literature, and wrote the manuscript. ST and KS revised the manuscript. MS provided important suggestions regarding the medical content. All authors read and approved the final manuscript.
